# A Novel TAF-Related Signature Based on ECM Remodeling Genes Predicts Glioma Prognosis

**DOI:** 10.3389/fonc.2022.862723

**Published:** 2022-04-27

**Authors:** Lin-jian Wang, Peipei Lv, Yongli Lou

**Affiliations:** ^1^Advanced Medical Research Center of Zhengzhou University, Zhengzhou Central Hospital Affiliated to Zhengzhou University, Zhengzhou, China; ^2^Department of Neurosurgery, Zhengzhou Central Hospital Affiliated to Zhengzhou University, Zhengzhou, China; ^3^Department of Radiology, Zhengzhou Central Hospital Affiliated to Zhengzhou University, Zhengzhou, China

**Keywords:** glioma, tumor-associated fibroblasts, ECM remodeling, tumor microenvironment, risk signature

## Abstract

The composition and abundance of immune and stromal cells in the tumor microenvironment (TME) dramatically affect prognosis. Infiltration of immunosuppressive tumor-associated fibroblasts (TAFs) is a hallmark of glioma. However, the mechanisms regulating TAF infiltration and the prognostic value of TAF-related genes in glioma remain unclear. In this study, we analyzed TAF infiltration by Estimating the Proportion of Immune and Cancer cells (EPIC) algorithm based on multiple glioma databases, including Glioblastoma and low-grade glioma merged cohort from The Cancer Genome Atlas (TCGA GBMLGG) cohort, the Chinese Glioma Genome Atlas (CGGA) #325 cohort, and the CGGA #693 cohort. TAF infiltration was increased in glioblastoma (GBM), and elevated TAF infiltration predicted poorer survival in gliomas. Gene enrichment analyses revealed that differentially expressed genes (DEGs) between low-grade glioma (LGG) and GBM were significantly enriched in the extracellular matrix (ECM) remodeling-related signaling, which may contribute to immune escape and resistance to immune checkpoint blockers (ICBs). To identify co-expression modules and candidate hub genes that may be associated with TAF infiltration, we performed weighted correlation network analysis (WGCNA) of DEGs. Afterward, univariate Cox regression, least absolute shrinkage and selection operator (LASSO) regression, and multivariate Cox regression analyses were performed to screen the positive prognostic hub genes. Finally, a high-efficacy prediction signature was constructed based on the expression of *S100A4, PLAUR*, and *EMP3*. The signature correlated with the abundance of TAF infiltration in glioma and was an independent risk factor for glioma. In conclusion, our findings suggested that the TAF-related signature was a valuable prognostic biomarker in glioma and provided potential targets for integrative therapy of gliomas.

## Introduction

Glioma is the most common malignant brain tumor in adults (1). Maximum surgical resection with radiotherapy and temozolomide chemotherapy remains the standard treatment, but median survival for glioblastoma (GBM) is less than 16 months (2). As one of the most concerning areas of nervous system tumors, the treatment of glioma remains a huge challenge. Recently, immune checkpoint blockers (ICBs) have made breakthrough achievements in the clinical treatment of various malignant solid tumors, and targeting tumor immune checkpoints to kill tumor cells has been regarded as a promising tumor therapy (3, 4). Unfortunately, preliminary results from CheckMate143, a phase III clinical trial in recurrent glioma, showed that Programmed cell death protein 1 (PD1) antibodies did not significantly improve outcomes (5–8).

Except for some individuals who benefited from immune checkpoint inhibitors, others remained resistant to the treatment. An increasing number of investigations have uncovered that the tumor microenvironment (TME) is particularly critical among the factors contributing to ICB resistance (9, 10). TME refers to the internal environment in which tumor cells live, including not only the tumor cells themselves but also surrounding immune and inflammatory cells, tumor-associated fibroblasts (TAFs), adjacent interstitial tissues, microvessels, and various cytokines and chemokines (11). Interestingly, an increasing number of studies suggested that TAFs, extracellular matrix (ECM) proteins, collagens, and tumor fibrosis were involved in resistance to immunotherapy (12–17). TAFs are mesenchymal cells that overdeposit fibrillar collagens and other profibrotic ECM components, therefore protecting tumor cells from ICBs or targeted therapy.

The most daunting obstacle to cancer treatment is overcoming the immunosuppressive TME. Unraveling the mechanisms regulating TAF infiltration presents an opportunity that is actively pursued to develop specific targeted therapies for glioma. In this study, we analyzed the TAF infiltration in glioma by EPIC algorithm. Positive prognostic genes *S100A4, PLAUR*, and *EMP3* that might affect TAF infiltration in glioma were screened by weighted correlation network analysis (WGCNA), univariate Cox, least absolute shrinkage and selection operator (LASSO), and multivariate Cox regression analyses and were used to construct an efficient risk signature. In conclusion, we revealed the relationship between TAF infiltration and malignancy of glioma, demonstrated the value of signature based on TAF-associated genes in predicting the prognosis of glioma, and facilitated the development of specific targeted therapies for glioma.

## Methods

### Datasets and Samples

The datasets for TCGA GBMLGG, Chinese Glioma Genome Atlas (CGGA) #325, and CGGA #693 cohorts were obtained from the University of California Santa Cruz (UCSC) Xena browser (https://xenabrowser.net/datapages/) (18) and the CGGA data portal (http://www.cgga.org.cn/) (19). After excluding cases with missing clinical data, the study finally included 647, 309, and 656 patients, respectively (**Supplementary Tables S1–S3**). Three GBM and three low-grade glioma (LGG) tissue samples were collected in the Zhengzhou Central Hospital Affiliated to Zhengzhou University. Clinical information for all tissue samples used in the study was listed in the supplementary material (**Supplementary Table S4**). The study was approved by the ethics committee of Zhengzhou Central Hospital Affiliated to Zhengzhou University, and informed consent was obtained from all patients.

### Immune Microenvironment Analysis and Differentially Expressed Gene Enrichment Analysis

Abundance of tumor-infiltrating immune and stromal cells in gliomas was calculated by the EPIC method on the TIMER2 platform (http://timer.cistrome.org/) (20). The differentially expressed genes (DEGs) (p < 0.05 and |log_2_FC| ≥ 1) were screened using the R package “limma” in TCGA cohort, CGGA #325 cohort, and CGGA #693 cohort, respectively (21). Gene Ontology (GO) analysis of DEGs was performed using R package “clusterProfiler”. Kyoto Encyclopedia of Genes and Genomes (KEGG) analysis was conducted using the KOBAS-i (http://bioinfo.org/kobas) (22).

### Construction of the Risk Signature

DEGs were analyzed using the R software package “WGCNA,” and genes with high connectivity in the tumor-infiltrating fibroblast significant module were identified as hub genes. Univariate, LASSO, and multivariate regression analyses were then performed to screen for positive hub genes significantly associated with overall prognosis. The risk score was calculated as follows:


$$Risk\;score=\sum_{i=1}^{n}\left(Coef_{i}*Exp_{i}\right)$$

### Immunohistochemical Staining

After baking, dewaxing, rehydration, antigen retrieval, and blocking, paraffin sections were incubated overnight with anti-PLAUR primary antibody (Abcam, ab218106, 1:250) at 4°C. After washing three times, the sections were incubated with biotin-labeled secondary antibody for 20 min at room temperature, followed by avidin-labeled Horseradish peroxidase (HRP) for 20 min at room temperature, and then stained with diaminobenzidine. Finally, the sections were counterstained with hematoxylin.

### Statistical Analysis

One-way ANOVA, Wilcoxon test, and t test were used to analyze the significance of differences in gene expression and immune cell infiltration. Univariate regression, LASSO regression, multivariate regression, and Kaplan–Meier analyses were performed using the R packages “glmnet” and “survival”. sinlge Receiver operating characteristic (ROC) curve was drawn using the R package “pROC”. All statistical analyses were performed using GraphPad Prism 8, R software, and SPSS, and p values <0.05 were considered statistically significant.

## Results

### Tumor-Associated Fibroblasts in Glioma

**Figure 1:**
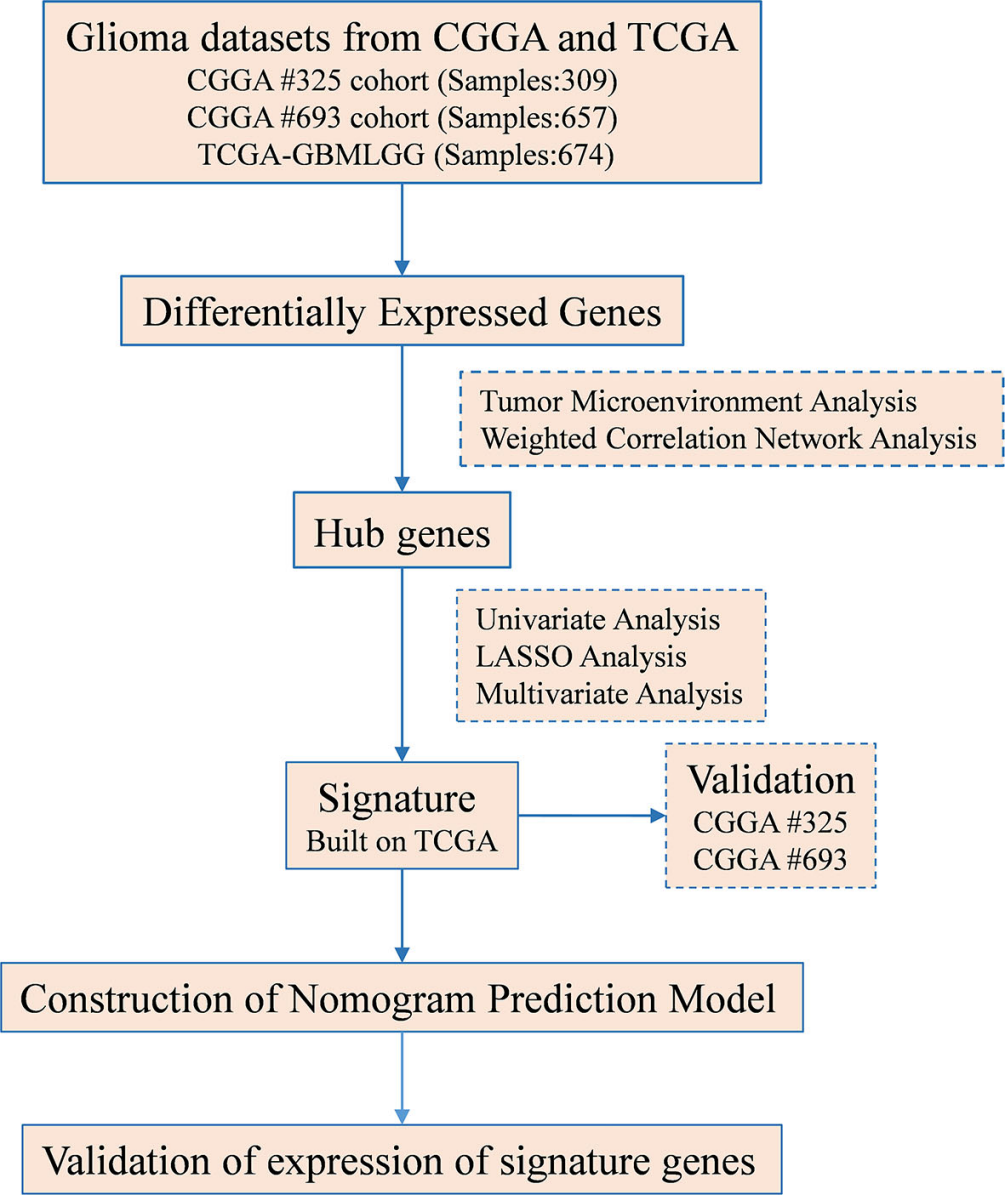
Flowchart of this study. TCGA, The Cancer Genome Atlas; CGGA, Chinese Glioma Genome Atlas.

**Figure 2:**
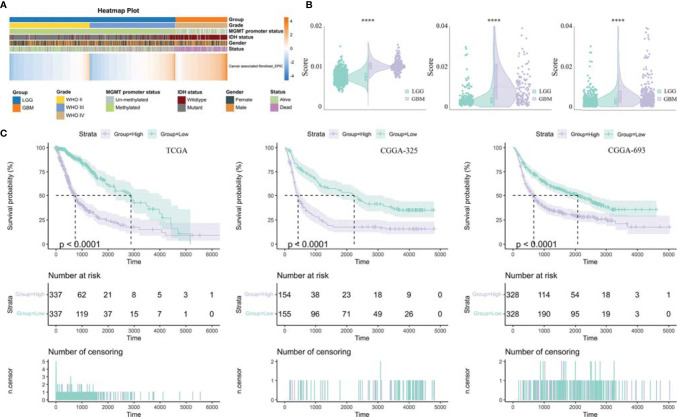
Analysis of tumor-infiltrating fibroblasts in glioma. **(A)** Heatmap was drawn to depict the TAF infiltration in glioma from TCGA GBMLGG cohort. **(B)** TAFs were increased in GBM relative to LGG in TCGA GBMLGG cohort, CGGA #325 cohort, and CGGA #693 cohort, respectively. **(C)** Kaplan–Meier curves displayed that increased TAF infiltration was associated with poor prognosis and lower survival rate of glioma. ****, p < 0.0001.

Previously, seven methods including CIBERSORT, CIBERSORT abs, quanTIseq, MCP-counter, TIMER, xCell, and EPIC have been evaluated for their accuracy in estimating different immune and stromal cells based on gene expression data. Considering the robust overall performance of the EPIC method in predicting TAF infiltration (23), we analyzed RNA Sequencing (RNA-seq) data from the CGGA #325, CGGA #693, and TCGA GBMLGG cohorts by this method to characterize TAFs in glioma (**Figures 1**, **2**). There were significant differences in TAF infiltration in different WHO grades of gliomas, with higher TAFs in high-grade gliomas (**Figure 2A**). Similarly, the infiltration of TAFs was significantly increased in GBM relative to LGG (**Figure 2B**). In addition, fibroblast infiltration was significantly associated with the prognosis of gliomas, with high infiltration predicting poorer overall survival (**Figure 2C**).

### Identification of Differentially Expressed Genes and Gene Enrichment Analysis

**Figure 3:**
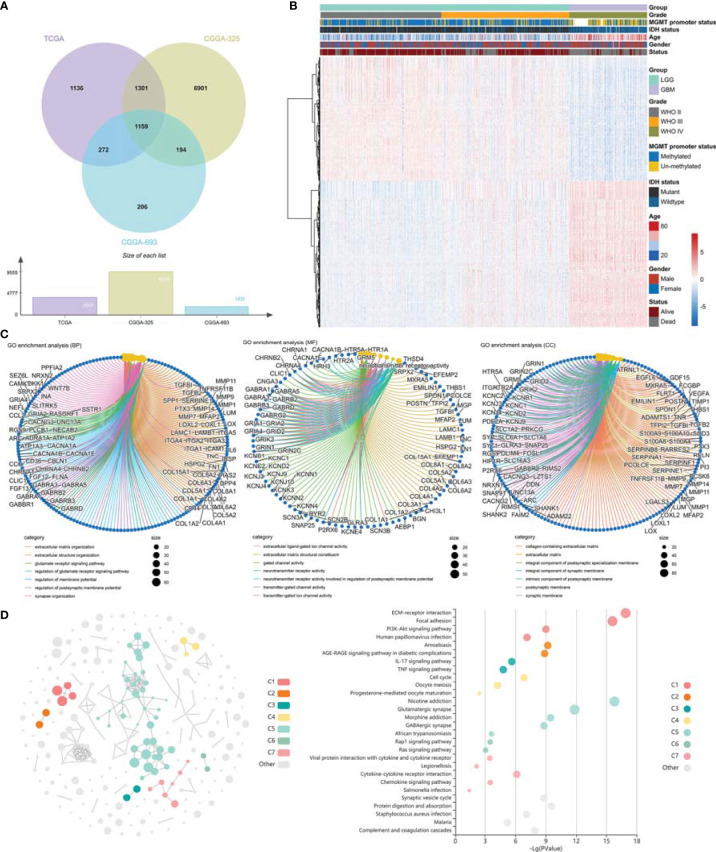
Identification of differentially expressed genes and gene enrichment analysis. **(A)** Venn diagram showed the differentially expressed genes in TCGA GBMLGG cohort, CGGA #325 cohort, and CGGA #693 cohort, respectively. **(B)** Heatmap displayed the expression profile of the 1,159 DEGs. **(C)** GO analysis of the differentially expressed genes. **(D)** KEGG analysis of the differentially expressed genes. GO, Gene Ontology; KEGG, Kyoto Encyclopedia of Genes and Genomes.

The DEGs (|log2FC| ≥ 1 and adjusted p < 0.05) between LGG and GBM were identified using R package “limma”. In this study, 3,868, 9,555, and 1,831 DEGs were screened out in TCGA GBMLGG cohort, CGGA #325 cohort, and CGGA #693 cohort, respectively, of which 1,159 DEGs were identifiable in all cohorts (**Figures 3A, B**). GO analysis of 1,159 target DEGs was performed using R package “clusterProfiler” (**Figure 3C**), and then KEGG analysis was conducted using the KOBAS-i (**Figure 3D**). GO analysis showed that the DEGs between LGG and GBM were mainly involved in the organization and remodeling of ECM (**Figure 3C**). KEGG analysis showed that the DEGs were significantly enriched in the ECM–receptor interaction pathway, focal adhesion pathway, Phosphatidylinositol 3 kinase-protein kinase B (PI3K-Ak) signaling pathway, Ras signaling pathway, cytokine–cytokine receptor interaction, and chemokine signaling pathway (**Figure 3D**). Therefore, ECM remodeling may be involved in glioma progression.

### Construction of the Risk Score Signature

**Figure 4:**
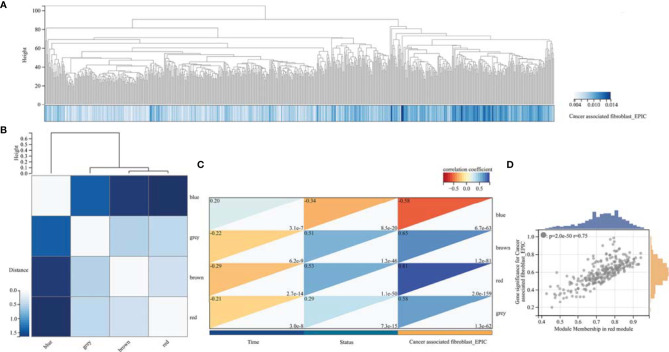
Weighted correlation network analysis. **(A)** Clustering of samples in TCGA GBMLGG cohort. **(B)** Cluster dendrogram of modules. **(C)** Module–trait relationships indicated that the red module was most related to TAF infiltration in glioma. **(D)** Scatter plot of correlation between GS and MM. GS, Gene significance; MM, Module membership.

**Figure 5:**
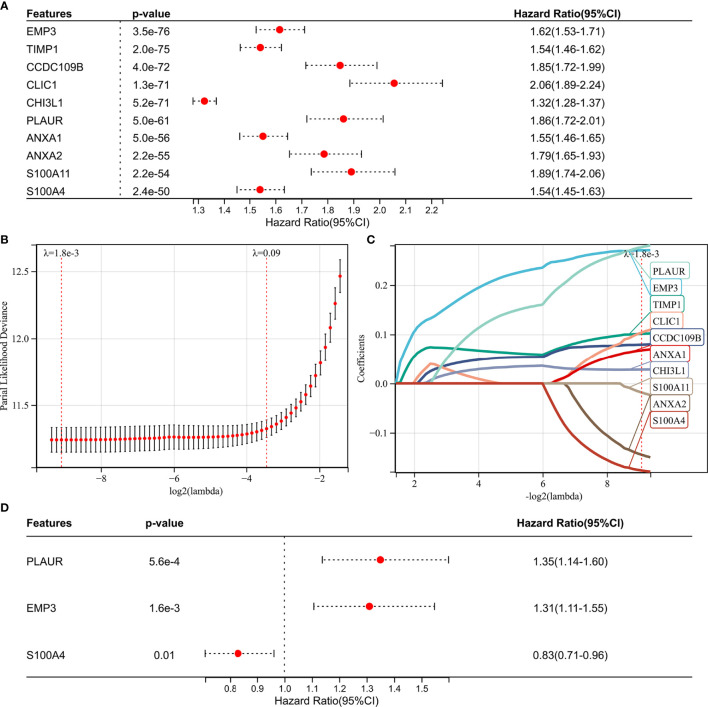
Screening of the prognostic genes in TCGA GBMLGG cohort. **(A)** Univariate Cox regression analysis of hub genes in TCGA GBMLGG cohort. **(B)** Partial likelihood deviance of different numbers of variables revealed by the LASSO regression model. **(C)** LASSO coefficient profiles of the positive hub genes. **(D)** Multivariate Cox regression analysis of hub genes in TCGA GBMLGG cohort.

**Figure 6:**
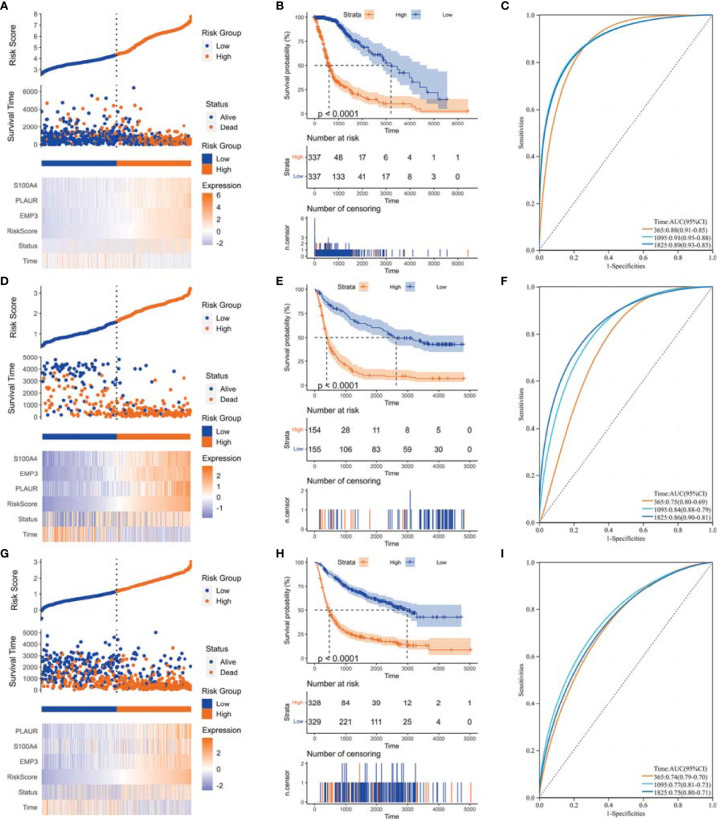
Construction of risk score signature. **(A)** Distribution of the risk score, survival status, and expression profile of the prognostic genes in the cohort of TCGA GBMLGG cohort, **(D)** CGGA #325 cohort, and **(G)** CGGA #693 cohort, respectively. **(B)** Kaplan–Meier curves displayed prognostic differences between high- and low-risk groups in the cohort of TCGA GBMLGG cohort, **(E)** CGGA #325 cohort, and **(H)** CGGA #693 cohort, respectively. **(C)** The ROC curves described the sensitivity and specificity of risk score in predicting OS at 1-, 3-, and 5-year time points in TCGA GBMLGG cohort, **(F)** CGGA #325 cohort, and **(I)** CGGA #693 cohort, respectively. ROC, Receiver operating characteristic.

We performed WGCNA to identify gene modules that are highly synergistically varied and to identify candidate hub genes based on the interconnectivity of gene modules and the association between gene modules and phenotypes (**Figure 4**). Four modules were identified from the co-expression network (**Figure 4B**), among which the red module was most associated with TAF infiltration (**Figures 4C, D**). Based on the cutoff criteria (|MM| > 0.9 and |GS| > 0.1), 10 genes with high connectivity in the clinically significant module were identified as hub genes. Univariate Cox regression analysis indicated that all the 10 hub genes were significantly associated with prognosis of glioma in TCGA GBMLGG cohort (**Figure 5A**). Subsequently, we performed LASSO and multivariate Cox regression analyses to analyze the 10 hub genes in TCGA GBMLGG cohort (**Figures 5B, C**). Finally, *S100A4, PLAUR*, and *EMP3* were identified as prognostic-related hub genes (**Figure 5D**) and were selected to construct the risk score signature in TCGA GBMLGG cohort (**Figure 6A**), CGGA #325 cohort (**Figure 6D**), and CGGA #693 cohort (**Figure 6G**), respectively. The survival of patients was analyzed using the R package “survival,” and finally, we observed a significant prognostic difference between the high-risk and low-risk groups (**Figures 6B, E, H**). We performed ROC analysis using the R package “pROC” at 1-, 3-, and 5-year time points to assess the sensitivity and specificity of risk score in predicting the survival of glioma patients and found that the predictive accuracy of the risk score was very high (**Figures 6C, F, I**).

**Figure 7:**
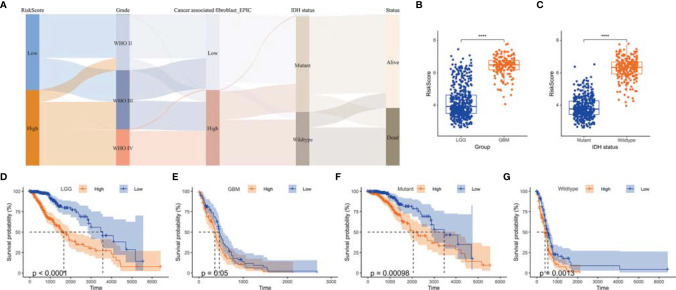
The value of risk scores in predicting prognosis. **(A)** Sankey diagram displayed the distribution of risk score, IDH status, survival status, WHO grade, and TAF infiltration of gliomas in TCGA GBMLGG cohort. **(B)** Boxplot showed that GBM had a higher risk score relative to LGG in TCGA GBMLGG cohort. **(C)** Boxplot showed that the risk scores of IDH wild-type group was higher than that of the mutant group in TCGA GBMLGG cohort. **(D-G)** Kaplan–Meier curves displayed prognostic differences between high- and low-risk groups in different WHO grades and IDH status subtypes. ****, p < 0.0001.

We then analyzed the relationship between the risk score and clinical phenotypes. A Sankey diagram was created to display the distribution of the survival status, WHO grade, Isocitrate dehydrogenase (IDH) status, risk score, and TAF infiltration of glioma patients (**Figure 7A**). As shown in **Figure 7B**, the risk score of glioma in GBM was significantly higher than that of the corresponding LGG subtype, and the IDH wild-type group had a higher risk score relative to the IDH mutant group (**Figure 7C**). In addition, the risk score signature also showed high prognostic value across different WHO grades and IDH status subtypes, and significant prognostic differences between high- and low-risk groups were observed in all subtypes (**Figures 7D–G**).

As the CGGA #693 cohort contained many recurrent gliomas (**Supplementary Table S3**), we analyzed the expression of prognostic genes and the risk scores in primary and recurrent subgroups, respectively. The expression of S100A4, EMP3, and PLAUR was increased in recurrent gliomas compared with primary gliomas (**Supplementary Figures S1A–C**) as were the risk scores of recurrent gliomas (**Supplementary Figure S1D**). Furthermore, recurrent gliomas had worse overall survival than primary gliomas (**Supplementary Figure S1E**), and the risk score signature also showed a high prognostic value in both primary and recurrent subgroups (**Supplementary Figures S1F, G**).

### The Relationship Between Risk Score and Tumor-Associated Fibroblast Infiltration

**Figure 8:**
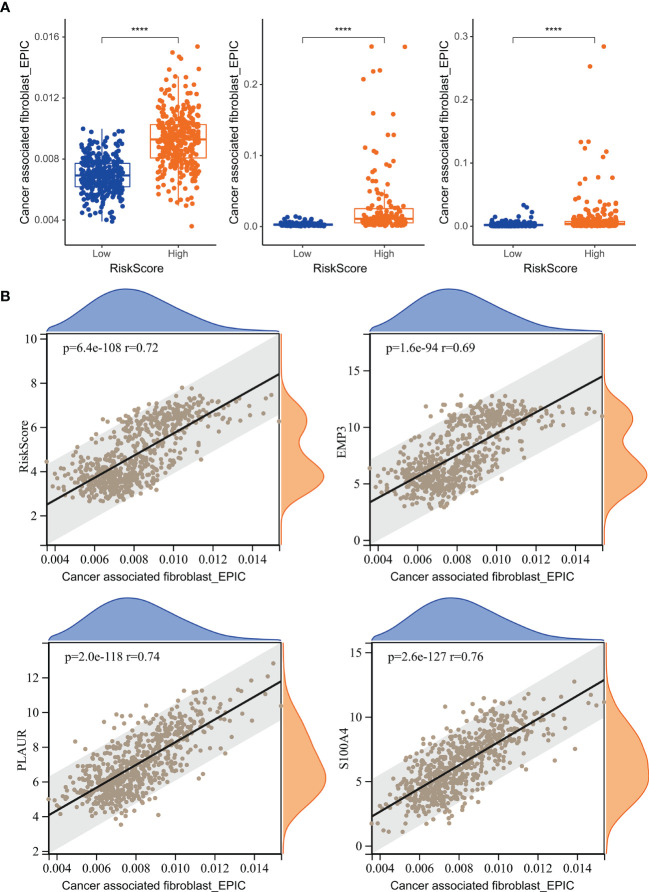
The relationship between risk score and TAF infiltration. **(A)** Boxplot showed that TAF infiltration in the high-risk group was increased relative to low-risk group in TCGA GBMLGG cohort, CGGA #325 cohort, and CGGA #693 cohort, respectively. **(B)** Correlation analysis showed that TAF infiltration was significantly associated with the risk score and the prognostic genes *EMP3, PLAUR*, and *S100A4*. ****, p < 0.0001.

We analyzed the relationship between risk score and TAF infiltration and found that the high-risk group had increased levels of fibroblast infiltration in all three cohorts (**Figure 8A**). In addition, there is a significant correlation between the risk score and TAF infiltration (**Figure 8B**). Prognostic-associated genes (*S100A4, PLAUR*, and *EMP3*) that constructed the risk signature were also significantly associated with TAF infiltration in gliomas (**Figure 8B**).

### Tumor-Associated Fibroblast-Related Signature Is an Independent Risk Factor for Glioma

**Figure 9:**
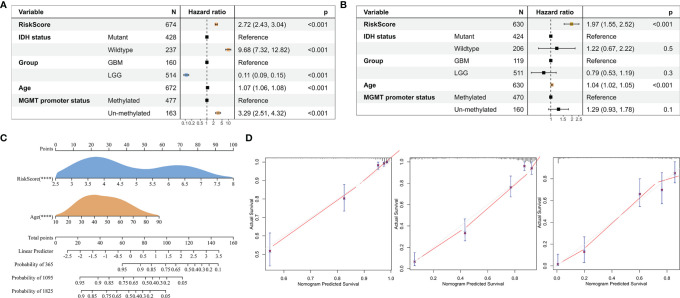
Risk score is an independent prognostic factor for glioma. **(A)** Univariate Cox regression analysis of the clinical phenotypes such as the risk score, age, MGMT promoter status, WHO grade, and IDH status. **(B)** Multivariate Cox analysis showed that the risk score was associated with the prognosis. **(C)** The nomogram was used to predict prognosis in patients at 1, 3, and 5 years in TCGA GBMLGG cohort. **(D)** Calibration curves showed the difference between the survival rate predicted by the model and the actual rate at 1, 3, and 5 years. ****, p < 0.0001.

To investigate whether risk score is an independent prognostic factor for gliomas in TCGA GBMLGG cohort, we performed univariate and multivariate Cox regression analyses, respectively. The results showed that the risk score was significantly correlated with prognosis and was identified as an independent prognostic factor for glioma (**Figures 9A, B**). We then built a survival nomogram prediction model based on independent prognostic parameters for gliomas in TCGA GBMLGG cohort (**Figure 9C**). The calibration curves displayed excellent agreement between observation and prediction at 1-, 3-, and 5- year time points (**Figure 9D**).

### Validation of the Expression of the Prognostic Genes

**Figure 10:**
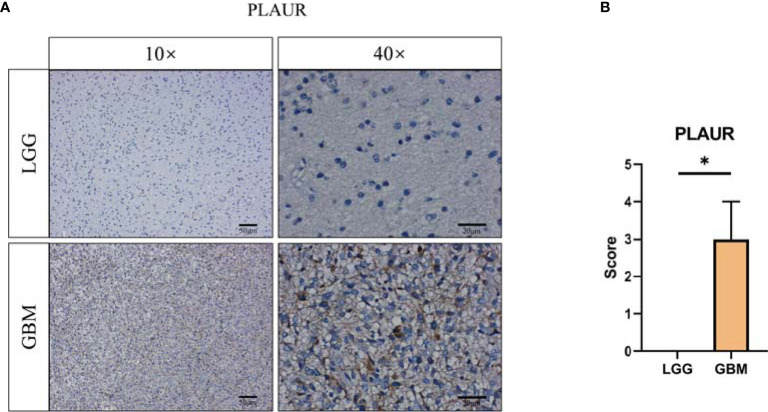
Validation of prognostic gene expression. **(A)** Immunohistochemical staining and **(B)** statistical analysis of PLAUR protein level between LGG (n = 3) and GBM (n = 3). *, p < 0.05.

To validate the expression of prognostic-associated genes that constructed the risk signature in glioma, we collected tissue samples resected from patients undergoing surgical treatment. Similar to the mRNA transcriptome results of TCGA GBMLGG cohort, CGGA #325 cohort, and CGGA #693 cohort, *PLAUR* expression was increased in GBM relative to LGG (**Figure 10**). Probably due to the limitation of the sample size collected in this study, only one gene was validated by immunohistochemical staining. Therefore, more samples may be required to verify the expression of *S100A4* and *EMP3*.

## Discussion

Glioma is considered to be one of the most devastating tumors in adults (24). The median survival of GBM remains poor with standard treatment of concurrent radiotherapy and chemotherapy after surgical resection (2, 25). Immunosuppressive TME has been shown to be associated with the progression of high-grade malignancies and resistance to treatment with ICBs (9, 10).

TAFs, as one of the most abundant cell types in the TME, can produce and overdeposit large amounts of ECM proteins and collagens and secrete a large number of chemokines, cytokines, and proteases, thus possessing various immunosuppressive functions and resistance to ICB treatment (26–28). Uncovering the mechanisms regulating TAF infiltration may facilitate the development of TAF-targeted immunotherapies, such as targeting ECM remodeling, reversing TAF phenotype, or depleting TAFs (29). In this study, we revealed that high TAF infiltration was significantly associated with poor over survival (**Figure 2**), and the TAF-related risk signature could accurately predict glioma prognosis. Gene enrichment analyses showed that the DEGs between LGG and GBM were significantly enriched in the ECM-related signaling pathway, which may contribute to ECM remodeling and resistant to ICBs (**Figure 3**).

TAFs are heterogeneous cell populations and may originate from many different cellular precursors (30). TAFs can be recruited and activated from normal resident tissue fibroblasts, largely depending on the TME (31, 32), as well as transdifferentiation from non-fibroblast lineages (33–37). Similar to previous studies (38, 39), *S100A4, PLAUR*, and *EMP3* were found to be associated with glioma progression to high grade (**Figures 3**, **5**) and were identified as the hub genes that may affect TAF infiltration in glioma (**Figures 4**, **8**). Therefore, the regulatory roles of these genes on the immune microenvironment should be further investigated.

*S100A4*, a well-known marker for defining TAFs in tumors (40), is involved in epithelial-to-mesenchymal transition (EMT) and endothelial-to-mesenchymal transition (EndMT) during the acquisition of a fibroblast phenotype in epithelial and endothelial cells (41, 42). S100A4^+^ TAFs contribute to immune evasion by ECM remodeling and recruiting macrophages (43, 44). *PLAUR* is aberrantly expressed in tumors due to genetic alterations and TME such as hypoxia. Coordination of tumor–ECM interactions, pericellular ECM proteolysis, and cell signaling underlies the critical role of *PLAUR* in tumor progression and survival (45). Lately, *EMP3* has been shown to mediate GBM‐associated macrophage infiltration to drive T-cell exclusion (46), but its impact on TAFs remains to be investigated. These functions, independent of regulation of proliferation, migration, and invasion, as well as potential regulation of TAFs, will provide potential targets for glioma immunotherapy.

## Conclusions

Overall, we revealed the relationship between TAF infiltration and malignancy of glioma, demonstrated the value of the TAF-related signature in predicting the prognosis of glioma, and provided potential targets for glioma immunotherapy.

## Data Availability Statement

The original contributions presented in the study are included in the article/**Supplementary Material**. Further inquiries can be directed to the corresponding author.

## Ethics Statement

The studies involving human participants were reviewed and approved by the medical ethics committee of the Zhengzhou Central Hospital Affiliated to Zhengzhou University. Informed consents were obtained from all individual participants included in the study.

## Author Contributions

L-jW and YL conceived and designed the experiments. PL contributed to data analysis. L-jW wrote the article. L-jW and YL approved the final version of the article.

## Funding

This work was supported by the National Natural Science Foundation of China (82101401).

## Conflict of Interest

The authors declare that the research was conducted in the absence of any commercial or financial relationships that could be construed as a potential conflict of interest.

## Publisher’s Note

All claims expressed in this article are solely those of the authors and do not necessarily represent those of their affiliated organizations, or those of the publisher, the editors and the reviewers. Any product that may be evaluated in this article, or claim that may be made by its manufacturer, is not guaranteed or endorsed by the publisher.

## Supplementary Material

Supplementary Figure 1 The value of risk scores in predicting prognosis. **(A-C)** Boxplot showed the expression of S100A4, EMP3, and PLAUR between primary and recurrent subgroups in the CGGA #693 cohort. **(D)** Boxplot showed that recurrent gliomas had higher risk scores relative to primary gliomas in the CGGA #693 cohort. **(E)** Kaplan-Meier curve displayed prognostic differences between primary and recurrent subgroups in the CGGA #693 cohort. **(F)** Kaplan-Meier curves displayed prognostic differences between high- and low-risk groups in primary and **(G)** recurrent gliomas, respectively. *, p< 0.05; **, p< 0.01; ***,p< 0.001, ****, p< 0.0001.

Supplementary Table 1 Clinical information on the gliomas involved in this study.
