# Supplementary figures and images for: A Novel TAF-Related Signature Based on ECM Remodeling Genes Predicts Glioma Prognosis
Source: Front Oncol. 2022 Apr 27;12:862723. doi: 10.3389/fonc.2022.862723 (PMC9093456; doi:10.3389/fonc.2022.862723)

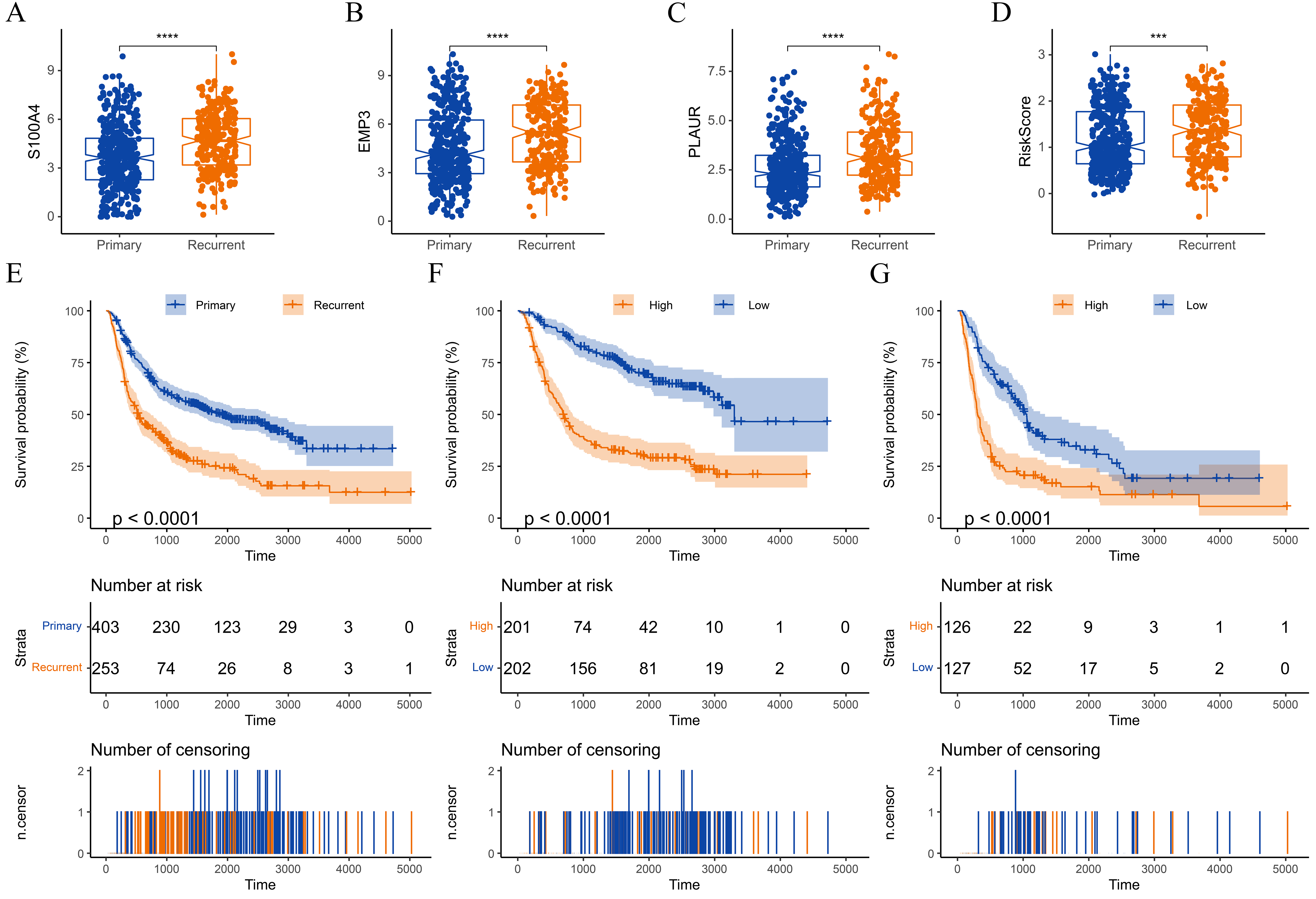

Supplement: Supplementary Figure 1 — The value of risk scores in predicting prognosis. (A-C) Boxplot showed the expression of S100A4, EMP3, and PLAUR between primary and recurrent subgroups in the CGGA #693 cohort. (D) Boxplot showed that recurrent gliomas had higher risk scores relative to primary gliomas in the CGGA #693 cohort. (E) Kaplan-Meier curve displayed prognostic differences between primary and recurrent subgroups in the CGGA #693 cohort. (F) Kaplan-Meier curves displayed prognostic differences between high- and low-risk groups in primary and (G) recurrent gliomas, respectively. *, p< 0.05; **, p< 0.01; ***,p< 0.001, ****, p< 0.0001. [file Image_1.tif]
